# Uncovering the Locus of Object-Context-Based Modulations in Depth Processing Using Repetitive Transcranial Magnetic Stimulation

**DOI:** 10.1523/ENEURO.0217-22.2022

**Published:** 2022-08-26

**Authors:** Nicole H. L. Wong, Dorita H. F. Chang

**Affiliations:** 1 Department of Psychology, The University of Hong Kong, Hong Kong; 2 State Key Laboratory of Brain and Cognitive Sciences, The University of Hong Kong, Hong Kong

**Keywords:** depth processing, object processing, transcranial magnetic stimulation

## Abstract

Neural responses of dorsal visual area V7 and lateral occipital complex (LOC) have been shown to correlate with changes in behavioral metrics of depth sensitivity observed as a function of object context, although it is unclear as to whether the behavioral manifestation results from an alteration of early depth-specific responses in V7 or arises as a result of alterations of object-level representations at LOC that subsequently feed back to affect disparity readouts in dorsal cortex. Here, we used online transcranial magnetic stimulation (TMS) to examine the roles of these two areas in giving rise to context–disparity interactions. Stimuli were disparity-defined geometric objects rendered as random-dot stereograms, presented in geometrically plausible and implausible variations. Observers’ sensitivity to depth (depth discrimination) or object identity (plausibility discrimination) was indexed while receiving repetitive TMS at one of the two sites of interest (V7, LOC) along with a control site (Cz). TMS over LOC produced results no different from TMS over baseline Cz (and prior no-TMS behavioral work). That is, depth sensitivity was higher for implausible versus plausible objects. Strikingly, TMS over V7 abolished differences in depth sensitivity for implausible versus plausible objects. V7 serves as a key locus in bringing stereosensitivity changes because of object context, perhaps by reweighing stereoscopic data en route to informing object–motoric interactions.

## Significance Statement

Stereosensitivity depends on object context (e.g., object identity/biological relevance). We applied repetitive transcranial magnetic stimulation (TMS) to two visual areas—depth-sensitive V7 and object-relevant lateral occipital complex (LOC)—to determine the source of this intriguing interaction. We show that TMS over V7 abolishes differences in stereosensitivity for different object types (i.e., plausible vs implausible objects). The effect is retained under LOC TMS. Our data suggest that V7 is a key site for serving context-based modulations in depth processing.

## Introduction

Binocular disparity, the difference between the left and right retinal images, is a powerful cue that allows the human brain to solve depth information from object structures. Previous work has established that disparity information is widely represented across both dorsal visual areas (V3A, V7) that are highly specific to the magnitude of disparity, and ventral visual areas including V4 and lateral occipital complex (LOC), the responses of which are much less specific (Welchman, 2016). Particularly, V3A and V7 appear to be sensitive to disparity-defined depth (Neri et al., 2004; Tyler et al., 2006) but are also involved in perceiving complex objects and curvature (Chandrasekaran et al., 2007 ; Georgieva et al., 2009). Beyond this, the intraparietal sulcus (IPS) is sensitive to 3D objects, which are disparity defined and motion defined, and the caudal intraparietal area is sensitive to surface slant (Sakata et al., 2005; Orban et al., 2006; Preston et al., 2008).

In ventral inferotemporal cortex, neurons are sensitive to disparity information (Uka et al., 2000) but only if it supports the perception of depth. They do not respond to anticorrelated random-dot stereograms (RDSs), in which the polarity of the dots in the left and right images are reversed and do not induce a depth impression (Janssen et al., 2003).

In recent work, it has become apparent that the particular areas engaged during the perception of disparity-defined depth depends on task demands, and whether the task entails segmenting disparity signals from noise or discriminating between two clear displays (Roe et al., 2007; Chowdhury and DeAngelis, 2008). Recent neuroimaging work from our laboratory seems to suggest an additional layer of complexity: depth responses are also modulated by object context. We found that both behavioral sensitivities and neural responses to disparity-defined depth change depending on the plausibility (Wong et al., 2020) or biological relevance (Chou et al., 2021) of the object presented, while other geometric and image-level properties are held constant. For example, the ability to extract depth targets from noise is better for implausible than plausible objects, and the behavioral phenomenon is paralleled by object context-related changes observed in V3, V3A, V7, and LOC (Wong et al., 2020). We speculated that such modulations may serve an ecological purpose of allowing better examination and identification of novel, unfamiliar stimuli. In some ways, the utility of such a system may be similar to that thought to underlie the “novel popup effect” (Johnston et al., 1990) in which novel objects are granted particularly high saliency relative to familiar objects, capturing attention and facilitating target detection.

Based on our earlier work (Wong et al., 2020), we were particularly interested in the roles of V7 and LOC in delivering object context-based influences on disparity sensitivity. Both have been extensively described in the disparity processing literature (Welchman, 2016; Rideaux and Welchman, 2019 ; Ng et al., 2021). Given the responsivity of both regions to disparity-defined depth, with LOC serving additional known roles in object analysis, it is unclear whether the context-based modulations of stereosensitivity we observed previously arose from early alterations of disparity responses in V7 or reflect changes in disparity readout subsequent to object-level assembly (i.e., feedback from LOC).

Previous work has documented well established connections between the depth-responsive regions (V3A, AIP) and the object-responsive areas (TEO; Webster et al., 1994; Borra et al., 2008). The circuitry is certainly present for established shape and object representations to receive from and feed back to the occipitoparietal regions. Connectivity work with an emphasis on disparity processing specifically is scarce; however, the involvement of a few very important dorsal visual areas has come to light. For example, the study by Ng et al. (2021) reported feedforward connections from V3A and V7, and feedback connections from V7 to V3A and from V3A to V1. Nevertheless, it is not entirely clear as to how object influences on disparity extraction may come about.

Here, we used repetitive transcranial magnetic stimulation (rTMS) to probe how V7 and LOC subserve modulations of disparity sensitivity based on object plausibility. We used an online stimulation protocol whereby high-frequency (10 Hz, 5 pulses) stimulation was delivered to the participant while performing one of two tasks: a depth discrimination task or a plausibility discrimination task (Mevorach et al., 2010 ; Chang et al., 2014). We predicted that if object–context modulations of disparity responses observed previously at LOC occur as an indirect consequence of modulations at V7, TMS over V7 would abolish the differences between depth sensitivities for plausible and implausible objects. Conversely, if context-based differences in disparity responses arise from LOC, TMS over LOC would abolish any differences between depth sensitivities for plausible and implausible objects. We further expect that disruption of LOC would worsen performance on a plausibility discrimination task more broadly, because of its known role in object analysis.

## Materials and Methods

### Participants

Sixty-four individuals (mean age, 22.73 years; SE, 0.57; 39 females) with normal or corrected-to-normal vision participated in the experiment. All participants provided written informed consent in line with procedures approved by the Human Research Ethics Board of the University of Hong Kong and followed methods that conform to the relevant guidelines and regulations. The study was conducted in accordance with the Declaration of Helsinki. All participants were screened for stereo deficits and visual acuity. They had indicated no history of neuropsychological disorders or any TMS and MRI contraindications. Forty-eight participants were stimulated during completion of the depth discrimination task (mean age, 23.31 years; SE, 0.64; 37 females). These participants were further subdivided into two stimulation groups (*N* = 24, LOC stimulation; *N* = 24, V7 stimulation). Sixteen additional participants were stimulated during a supplementary plausibility discrimination task (mean age, 22.81 years; SE, 1.24 years; 11 females). Again, these participants were subdivided into two stimulation groups (*N* = 8, LOC stimulation; *N* = 8, V7 stimulation). Final sample sizes were retrieved based on a power analysis in consideration of effect sizes from previous behavioral, fMRI, and rTMS depth-related studies (Preston et al., 2008; Chang et al., 2014; Wong et al., 2020), and object-related TMS work (Chouinard et al., 2017).

### Experimental procedures

The experiment consisted of the following two stages: (1) MRI localization of brain areas; and (2) three TMS sessions completed across 3 separate days.

### fMRI—localization of brain areas

#### Image acquisition

Because of a changeover of MRI equipment at the University of Hong Kong, participants were scanned across two scanners. fMRI data (i.e., functional localizers) for the first 48 subjects were acquired using a 3 T Achieva TX MR scanner (Philips Healthcare) with a 32-channel, phase-array (whole) head coil. Blood oxygenation level-dependent signals were measured with an echoplanar sequence (2 × 2 × 2 mm; TR = 2000 ms; TE = 30 ms; 30 slices). The remaining 16 participants were scanned with a Sigma Premier scanner (GE Healthcare) and a 48-channel phase-array coil (echoplanar sequence, 2 × 2 × 2 mm; TR = 2000 ms; TE = 30 ms; 58 slices). A high-resolution (1 mm^3^) anatomic scan was acquired for each participant.

#### Identification of regions of interest

For each participant, retinotopic visual areas (V1, V2, V3, V3A, V3B, V4, V7), LOC, and hMT+ were identified using well established procedures previously described in the study by Wong et al. (2020).

Stimuli were presented in E-Prime 1.1 (Psychology Software Tools) via an E-Sys presentation system (Philips Medical Systems) for the first 48 subjects (i.e., before scanner upgrade), placed at the back of the bore, and viewed using a front-surfaced mirror mounted on the head coil. After the upgrade, the final 16 subjects viewed stimuli presented using a PROpixx DLP LED projector (VPixx Technologies) that was side projected to a mirror placed behind the bore. The mirror image was then projected to a second screen placed directly at the base of the bore. Participants viewed the stimuli through a front-surfaced mirror.

Head movement was limited by foam padding within the head coil. All scan protocols and screening of participants adhered to the strict restrictions of the Imaging Unit. One fMRI session lasted 45 min.

#### Imaging data analysis

MRI data were processed using Brain Voyager 20.6 (Brain Innovations). The initial two volumes of each functional run were discarded to eliminate the effects of startup magnetization transients in the data. Functional data were preprocessed using slice-time correction, 3D motion correction, high-pass filtering (three cycles/run), and linear trend removal. Functional images were aligned with each participant's anatomic scan. To prepare for the TMS sessions, functionally defined regions of interest (ROIs) were overlayed on to the T1 image (in native space) and imported into Brainsight (Rogue Research) for neuronavigation.

### rTMS procedures

#### Stimuli

**Figure 1.**
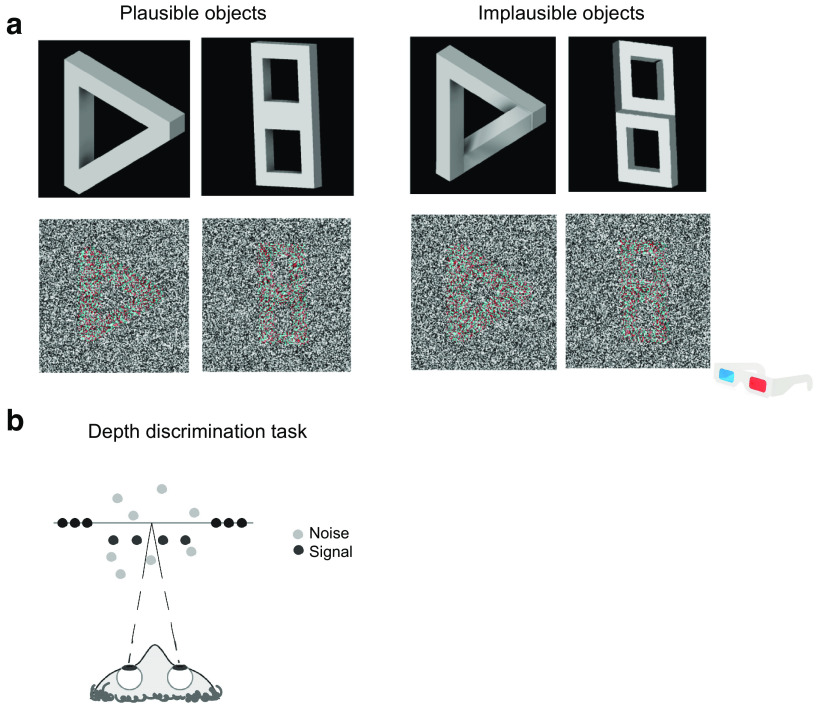
Plausible and implausible variants of the 3D objects used in the current study. ***a***, Depth maps (top row) were rendered into RDSs (bottom row). The geometry of the stimuli for the depth discrimination task and plausibility discrimination task. ***b***, Participants were asked to judge whether the target was in front of or behind a zero-disparity reference plane (depth discrimination task). Note that grayscale images were shown via a mirror stereoscope in the experiment proper (i.e., red-cyan anaglyphic stimulus examples are shown here for greater clarity).

Stimuli consisted of two classes of 3D objects (triangle and cube), each with a physically plausible and implausible variation, rendered as RDSs. 3D stimuli were first generated using Inventor Studio 2018 (Autodesk; Fig. 1*a*) and were then defined in terms of depth maps. To match low-level features between plausible and implausible objects, (1) the beam width and surface areas were equivalent across the two variations of objects and (2) the overall disparity across both object variations were equivalent. From the depth maps, RDSs were finally generated by computing the corresponding horizontal displacement from the gray-level intensity maps.

The RDSs were presented using MATLAB (MathWorks) with extensions from the Psychophysics Toolbox (Brainard, 1997; Pelli, 1997). Dots of the RDSs were randomly black and white and had a density of 40 dots/degree^2^, with each dot subtending 0.023°. Each RDS was presented on a black background and subtended 7.4° × 7° in size. All objects had a maximum disparity of 3.31 arcmin. The RDS was additionally surrounded by a grid of black and white squares (size, 0.44°) to assist vergence. The RDSs were presented on the left and right halves of the monitor, which were viewed through the silver-fronted mirrors.

#### Apparatus: stimulus and TMS delivery

Stimuli were presented on a mirror stereoscope in which each eye viewed the left or right half of a 24 inch monitor (resolution, 1920 × 1080; refresh frequency, 60 Hz) through silver-fronted mirrors mounted and oriented at 45° angles. The viewing distance was 65 cm, as stabilized with a chinrest. rTMS pulses were delivered using a Rapid^2^ Plus Stimulator equipped with a 70 mm figure-of-eight coil (Magstim). Neuronavigated TMS was guided using a Polaris Infrared Tracking System (Northern Digital Instruments).

#### Stimulation sites and design

**Figure 2.**
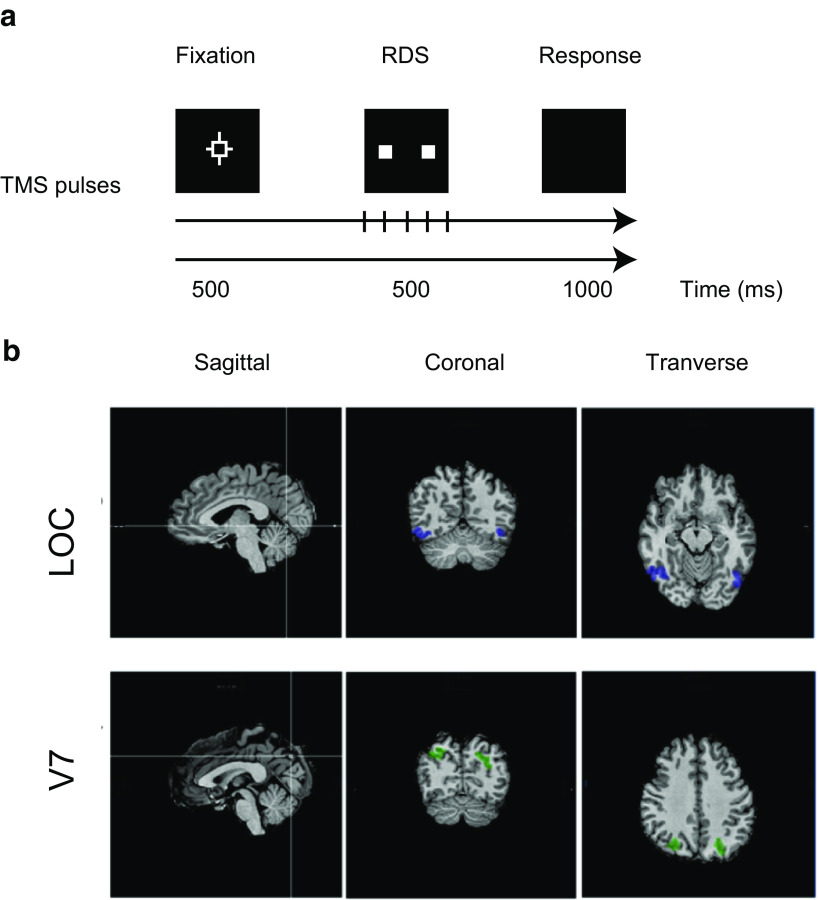
Experimental design and TMS stimulation sites. ***a***, The illustration shows the flow of one experimental trial: a 500 ms nonius-type fixation was followed by a 500 ms disparity-defined object (RDS). Participants responded within 1 s after stimulus offset. TMS pulses were delivered concurrent with stimulus onset (five 10 Hz pulses at 60% of the maximum output of the stimulator). ***b***, Sample ROIs of LOC (top row) and V7 (bottom row) of one representative participant.

Stimulation sites (V7, LOC) were localized via fMRI retinotopic and object localizers. For control site (Cz), the location was identified for each participant using TMS/EEG caps with nodes marked according to the 10–20 EEG coordinate system. Cz has been a commonly selected baseline site in prior vision-relevant TMS work (Nuding et al., 2009; Pitcher et al., 2009; Mevorach et al., 2010; Chang et al., 2014). The TMS coil, with its handle pointing upward or posteriorly (for Cz stimulation), was held tangentially to the surface of the skull to minimize the distance between the coil and the cortex (Ulmer and Jansen, 2010). During each stimulation session, the coil position was monitored in real time to ensure that the center of the figure-of-eight coil was no more than 3 mm away from the center of the ROI. The coil was repositioned if any subject movements caused deviations during trial runs. TMS was delivered at 10 Hz (five pulses synchronized with stimulus onset) with a fixed intensity of 60% of the stimulator’s maximum output. We elected to proceed with a fixed intensity protocol for two reasons: First, it is unclear that an intensity derived from motor cortex thresholding will translate to comparable effectiveness when applied over visual areas, given variances in tissue thickness across the surface. Second, a fixed intensity stimulation protocol at 60% maximum output has been shown to be both safe and able to elicit reliable effects (Mevorach et al., 2010 ; Chang et al., 2014). Five pulses were delivered on each trial, yielding a total of 520 pulses per run (104 trials), and 1040 pulses per session. Object stimuli were presented for 500 ms with TMS onset concurrent with the onset of stimulus presentation (Fig. 2). The intensity, frequency, and number of pulses aligned with safety limits described in studies by Rossi et al. (2009) and Wassermann (1998).

Each participant was tested across three sessions on 3 d. Individuals were stimulated over a single ROI (V7/LOC), on the left and right sides along with Cz, across the three sessions. TMS was administered to one ROI located in the left or right hemisphere, or over Cz while the participant completed the task. Order of stimulation sites was counterbalanced across participants. One TMS session took 60 min, including setup and calibration. Actual stimulation time was 10 min (2× 5 min runs).

#### Tasks

##### Depth discrimination task

The RDS was presented on a black background. During each run, the signal of the stimulus was adjusted according to a 3-down/1-up staircase procedure to measure the percentage of signal required for subjects to attain a task accuracy of 75% correct (initial threshold, 95; initial step size, 20; Levitt step rule). According to the Levitt rule, for the 1st, 3rd, 7th, and 15th reversals, the step size was reduced by two (Wetherill and Levitt, 1965). Each trial began with a 500 ms nonius-type fixation, followed by the RDSs presented for 500 ms. Participants were instructed to indicate the depth position of the object (in front/behind a reference plane) by keyboard press within 1 s after the stimulus had disappeared from the monitor. The next trial started immediately when the 1 s response period ended. Each run consisted of one object type (e.g., plausible), interleaving two variations of object class (equivalent number of triangle and cubes trials), yielding a total of 104 trials. Each participant completed two runs.

##### Plausibility discrimination task

The RDS was presented on a black background at 100% coherence signal. In each trial, participants were asked to indicate whether the object presented was a plausible or an implausible object by keyboard press. All other procedures and parameters were identical to those described for the depth task.

## Results

### Depth discrimination during TMS

We first conducted a 2 (plausibility: plausible, implausible objects) × 2 (group: LOC, V7) repeated-measures ANOVA (rmANOVA) to verify that both the LOC stimulation and V7 stimulation groups had comparable initial sensitivities during baseline (Cz). Results indicated a main effect of plausibility (*F*_(1,46)_ = 9.95, *p *=* *0.003, ηp^2^ = 0.178), but no main effect of group (*F*_(1,46)_ = 0.144, *p *=* *0.706, ηp^2^ = 0.066) or interaction between object plausibility and group (*F*_(1,46)_ = 0.216, *p *=* *0.644, ηp^2^ = 0.005). There was a significant difference between the thresholds for the two object variations (i.e., plausible and implausible objects) for the LOC group (*t*_(23)_ = 2.23*, p *=* *0.036, *d *=* *0.455) and the V7 group (*t*_(23)_ = 2.30*, p *=* *0.031, *d *=* *0.470) during stimulation over Cz. Consistent with the study by Wong et al. (2020), the thresholds for implausible objects were lower than those for plausible objects.

We proceeded to investigate the effect of TMS by means of a 2 (plausibility) × 2 (hemisphere: left, right) × 2 (group: LOC, V7) rmANOVA after normalizing data to baseline. To do so, we subtracted the thresholds obtained during Cz TMS from those obtained during TMS over the left and right ROIs respectively (Table 1). The analysis indicated a significant interaction between hemisphere and group (*F*_(1,46)_ = 4.30, *p *=* *0.044, ηp^2^ = 0.085). Follow-up independent *t* tests comparing normalized thresholds obtained during TMS over left and right ROIs between the LOC and V7 groups indicated that the thresholds were significantly higher during stimulation over left V7 relative to those during stimulation over left LOC (*t*_(46)_ = −2.26, *p *=* *0.029, *d* = −0.65; two tailed). The thresholds on the right hemisphere were not significantly different between the LOC and V7 groups. Additional follow-up paired *t* tests comparing thresholds within groups, but between hemispheres, indicated a significant difference between left and right thresholds for the V7 group (*t*_(23)_ = 1.76, *p *=* *0.046, *d *=* *0.358; one tailed) but not the LOC group (*t*_(23)_ = −1.18, *p *=* *0.12, *d* = −0.241; one tailed).

**Figure 3.**
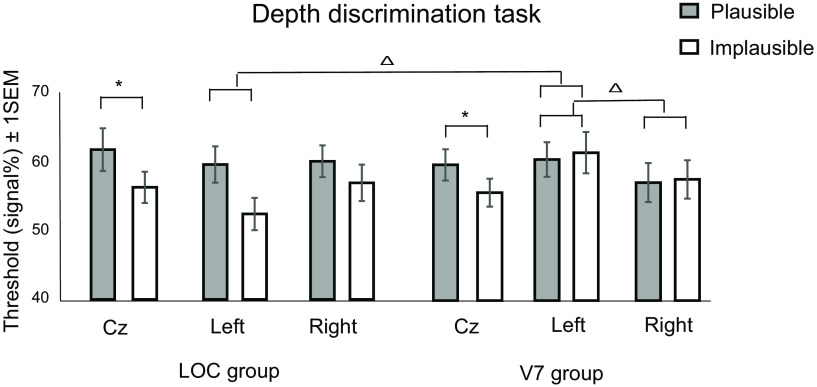
Behavioral depth discrimination thresholds under the administration of TMS. Depth thresholds (raw, unnormalized data) for both plausible and implausible objects obtained during the administration of TMS over the left and right hemispheres of the following three regions of interest: Cz, LOC, and V7. Depth thresholds are presented for the LOC group and the V7 group. Error bars represent ±1 SEM (*N* = 24/group). Asterisks indicate significant differences between thresholds for plausible versus implausible objects at Cz for both V7 and LOC groups. Triangles indicate significant comparisons between normalized depth thresholds for left V7 versus left LOC, and left V7 versus right V7.

To better assess the effects of TMS over the main sites of interest relative to Cz, we ran an additional 2 (plausibility) × 3 (site: left, right, Cz) × 2 (group: LOC, V7) ANOVA that indicated a main effect of plausibility (*F*_(1,46)_ = 9.34, *p *=* *0.004), and a significant interaction between plausibility and group (*F*_(1,46)_ = 4.63, *p *=* *0.037). The three-way interaction was not significant. Follow-up *t* tests for the two-way interaction indicated that thresholds were significantly lower for implausible objects versus plausible objects for the LOC stimulation group, but not the V7 stimulation group. While it would be easy here to conclude from looking at this analysis alone that TMS was ineffective, we argue that the apparent abolishment of the “plausibility” effect in the V7 stimulation group (which, on inspection of Fig. 3, is clearly carried by stimulation over left and right V7, and appears to be preserved over Cz) at least lends some weight to the effects reported in the earlier differenced analysis.

**Table 1:** Statistical table

Analysis	Figure	Data structure	Type of test	Observed power
Depth discrimination task (to verify that both LOC and V7 groups had comparable baselines)	Figure 3	Assumed normal	Repeated-measures ANOVA	0.870
Depth discrimination task (to measure the effects of TMS)	Figure 3	Assumed normal	Repeated-measures ANOVA	0.528
Plausibility discrimination task	Figure 4	Assumed normal	Repeated-measures ANOVA	0.460

### Object plausibility discrimination during TMS

**Figure 4.**
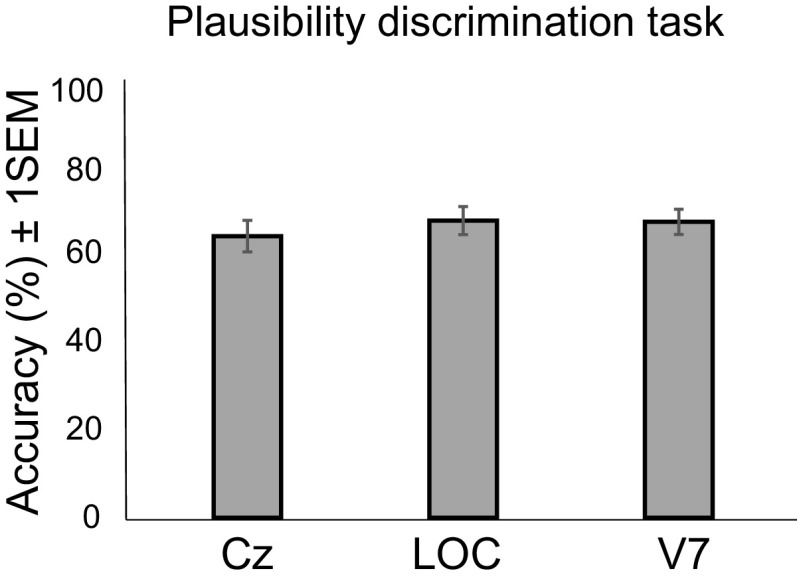
Behavioral performance in the plausibility discrimination task under TMS. Performance, indexed in terms of percentage-correct accuracies for distinguishing the physical plausibility of the object presented. Error bars represent ±1 SEM (*N* = 16).

We further compared accuracies for judging the plausibility of objects under Cz, LOC, and V7 stimulation (Cz: mean, 64.72; SD, 28.87; LOC: mean, 68.28; SD, 25.74; V7: mean, 68.01; SD, 23.13; Fig. 4) using an rmANOVA that indicated accuracies were comparable across all sites (*F*_(2,30)_ = 2.486, *p *=* *0.10, ηp^2^ = 0.142).

## Discussion

We (Wong et al., 2020) had previously demonstrated that behavioral sensitivities to disparity-defined depth can be altered depending on the object context (plausibility), and that this phenomenon was well correlated with changes in neural responses in V7 and LOC. Here, we sought to understand which of the two loci is key for delivering object–context-based changes in depth sensitivity.

Consistent with our initial demonstrations (Wong et al., 2020), we found that baseline (Cz) depth thresholds were higher for implausible objects relative to plausible objects. This was true in both groups of participants. More revealing, we found that TMS over LOC did not bring about any changes—that is, LOC TMS did not diminish the differences between depth thresholds for plausible and implausible objects. By contrast, V7 TMS resulted in comparable levels of thresholds between the two object types (plausibility). While our findings will benefit from future empirical validation, our current data suggest V7 as the key site serving context-based modulations in stereosensitivity, and that the effects observed previously at LOC in our fMRI report may have occurred as an indirect consequence of modulations at V7. We consider the consequence of TMS delivered over LOC versus V7 in turn.

### TMS over LOC

If the context-based differences in depth responses arise following object analyses in LOC, and are then subsequently delivered to V7, differences between depth sensitivities for plausible and implausible objects should diminish with LOC stimulation. This was not the case in our data. Disruption of LOC produced results no different from those of Cz TMS (baseline; i.e., depth sensitivities for implausible objects were higher than those for plausible objects). The lack of changes in response to TMS could be attributed to the following two possible reasons: (1) contextual modulation does not happen in LOC; and (2) stimulating LOC in a lateralized manner (i.e., solely right or left) is not sufficient to induce measurable perceptual changes. The present task does not allow verification of the activity of LOC during stimulation, nor can we speculate the extent of the LOC not under stimulation might have been affected based on the data. The lack of observable accuracy changes in our alternate (plausibility differentiation) task during LOC TMS suggests that disruption of LOC might be insufficient for disrupting object analysis here.

Contrary to expectations, the object plausibility discrimination accuracies remained robust after LOC stimulation. Previous TMS studies have demonstrated that stimulation over the right LOC leads to worsened object discrimination performance (Pitcher et al., 2009 ; Mullin and Steeves, 2011), and longer reaction time when shape discrimination was asked of the participants (Ellison and Cowey, 2006). However, it is worth noting that the aforementioned TMS studies invariably used 2D stimuli, in addition to slightly different TMS protocols (e.g., higher intensity, use of sham TMS instead of vertex TMS as a control, or even TMS pulses at different frequencies). Some or all of these differences may explain why we were unable to abolish or diminish object discrimination performance here. Indeed, it was important for us to keep constant the TMS stimulation train and the stimuli between our two tasks so that the only difference is the perceptual judgment required of the subject.

### TMS over V7

If context-based modulations of disparity responses rely on LOC representations, V7 TMS should not abolish such modulations (i.e., differences between depth sensitivities for plausible and implausible objects). However, if the effect observed previously in LOC occur as an indirect consequence of modulations at V7, V7 TMS should act to reduce the differences between depth sensitivities for plausible and implausible objects.

Unlike LOC TMS, our data showed that V7 stimulation indeed acted to abolish differences in thresholds between object variations. Could V7 be where the reweighing of depth signals because of object status is done? If so, how might this fit with the previous established role of the posterior parietal cortex, in disparity-noise segregation; and how might it fit eventually with object analysis?

Solving a perceptual task requires at least the following three elements: (1) a segregation of the stimulus, (2) a readout of the stimulus features, and (3) a decision-making unit (Dosher and Lu, 2005). In nonstereoscopic domains, visuoparietal regions have been shown to contribute to signal readouts and “flexible” computations (Li et al., 2012; Swaminathan and Freedman, 2012). Birman and Gardner (2019) tested the flexibility of readout systems by inserting “catch trials” in a motion visibility task in which participants had to distinguish which of two random-dot stimuli patches had higher contrast or coherence. During the catch trials, a cue was presented after stimulus offset, which indicated to the participants that they had to respond with respect to a different feature. Critically, participants could still perform these trials accurately. Apparently, while the original perceptual decisions were ready by the time the extra cue was presented, a rapid re-assembly of visual information relevant to the context could be achieved to adjust to a new judgment. The findings suggest that decisions may be subject to continuous updating of visuospatial data, perhaps arriving via visuoparietal cortex. Consistent with this, previous neuroimaging work involving stereoscopic depth judgments has similarly revealed that the IPS is involved in the readout of feature representations and evidence accumulation while making perceptual decisions (Patten and Welchman, 2015).

To the best of our knowledge, no other attempts at disentangling depth-processing mechanisms using online rTMS have been published in the last 2 decades except for the study by Chang et al. (2014), which reported poorer extraction of depth signal from noise during IPS stimulation. Interestingly, here, we observed that the thresholds collapsed across plausibility during TMS over left and right V7 were largely not different from those obtained during Cz TMS. The above suggests a potential segregation of the roles of the IPS more broadly versus the posterior V7 region: the IPS, serving signal segregation more broadly; and V7, perhaps serving a rapid reweighing of visual information in accordance with visual context. Of course, it is necessary to acknowledge that with TMS, targeted stimulation of V7 (here) or the more anterior portions of IPS (previous work) is still likely to elicit a bleeding of stimulation to the adjacent region, because of their proximity.

In the current data, LOC stimulation did not significantly affect object-based modulations of depth responses. We therefore speculate that the LOC engagement observed in our previous fMRI work (Wong et al., 2020) arrives following reweighing depth responses in the IPS/V7, to facilitate further object interactions. Previous neuroimaging work has reported that circuitry between ventral intraparietal sulcus (VIPS)/V7 and intraparietal areas, which use visual information for motor planning and grasping (Kaas, 2012; Baker et al., 2018). Particularly, the lateral intraparietal area has been implicated in disparity- and motion-related perceptual decisions during noise manipulations (Roitman and Shadlen, 2002 ; Gold and Shadlen, 2007). The anatomic situation of V7 (including its extensive connections to motoric and decision areas), along with its apparently sensitivity to object context makes it well positioned to contribute to motoric planning. Crucially, the LOC is also well connected with lateral intraparietal sulcus and VIPS, and can therefore relay any object information that could further assist in motoric planning–object interaction.

Our current data suggest that V7 is the key site that governs the reweighing of depth information based on object context. Future work may explore the intricacies within the depth processing network that incorporates object-level knowledge in a paradigm that explicitly allows the testing of connectivity patterns. It would be interesting to investigate how V7 is involved in other context-based modulations in depth processing, and whether its role holds true in other aspects of depth processing (e.g., shape, feature discriminations).

## Synthesis

Reviewing Editor: David Franklin, Technische Universitat Munchen Decisions are customarily a result of the Reviewing Editor and the peer reviewers coming together and discussing their recommendations until a consensus is reached. When revisions are invited, a fact-based synthesis statement explaining their decision and outlining what is needed to prepare a revision will be listed below. The following reviewer(s) agreed to reveal their identity: David Pitcher, Rossella Breveglieri.

Your manuscript has been reviewed by two experts in the field. They both have interest in the manuscript and think that this is an important test of the causal role of two brain areas in depth sensitivity. However, we all see significant issues with the level of explanation and details provided within the manuscript. We think that the current manuscript lacks sufficient details to perform an accurate assessment. Nevertheless, there appears to be sufficient interest to see a revised version of the paper in order to make an informed decision at that time. I encourage you to revise your manuscript after addressing each of the concerns raised by the reviewers.

General comments from the reviewers.

The manuscript investigates the specific causal role of areas V7 and LOC in context-dependent stereo-sensitivity. The main finding was that V7, but not LOC, is causally involved in context-dependent stereo-sensitivity. However, the current manuscript lacks details of the numbers of subjects and the exact experimental design, along with concerns over the data presentation/normalization, statistics, and issues relating to the Cz condition. Our major concerns lie with the issue that we are unable to determine the exact design used for the study, and where the choice of numbers of participants comes from, particularly due to the large difference in participants numbers for different experiments. Specific comments follow below.

Methods:

It appears that this is a between subjects design (LOC and V7 groups) but this is not made explicit and it is unclear how many subjects are in each group. A clearer description of the design is needed to make a full assessment.

Was there a power analysis to justify the sample size? Furthermore, was there a reason for the differences in the two sample sizes of the two experiments (N=48 and 16)?

Authors should reference to other existing literature to justify the Cz control condition.

Authors should justify why they used a fixed intensity of 60% of the stimulator’s maximum output instead of a percentage of the individual resting motor threshold.

Authors should mention the Declaration of Helsinki, as in every paper with human participants.

Results:

It is unclear whether Figure 3 shows the raw data for the TMS to V7 and LOC groups. If the data is normalized by subtracting from baseline the raw data needs to be displayed as well.

Why do the authors not put Cz in the levels of factor GROUP in the ANOVA. Related to this, it is unclear how the authors demonstrate the differences from the baseline.

Please specify better the variable tested with the first ANOVA.

In the first ANOVA was there any main effect of group? Please specify.

Figure 3 please indicate the significant comparisons.

Please be consistent in naming the tasks all across the paper.

Please be consistent in naming the areas. LO or LOC???

Minor concerns:

Significance statement: please change ‘V7 TMS’ with TMA over V7.

Introduction: what is RDS??

Introduction, this sentence ‘The limited connectivity work with an emphasis on disparity processing specifically, have focused on a few but very important dorsal visual areas.’ should be rephrased.
